# MicroRNA-7 Deficiency Ameliorates the Pathologies of Acute Lung Injury through Elevating KLF4

**DOI:** 10.3389/fimmu.2016.00389

**Published:** 2016-10-07

**Authors:** Juanjuan Zhao, Chao Chen, Mengmeng Guo, Yijin Tao, PanPan Cui, Ya Zhou, Nalin Qin, Jing Zheng, Jidong Zhang, Lin Xu

**Affiliations:** ^1^Department of Immunology, Zunyi Medical College, Guizhou, China; ^2^Department of Medical Physics, Zunyi Medical College, Guizhou, China

**Keywords:** miR-7KD, acute lung injury, immune cells, cytokines, KLF4

## Abstract

Recent evidence showed that microRNA-7 (miR-7) played an important role in the pathologies of lung-related diseases. However, the potential role of miR-7 in acute lung injury (ALI) still remains poorly understood. Here, we assessed the effect of miR-7 deficiency on the pathology of ALI. We, first, found that the expression of miR-7 was upregulated in lung tissue in murine LPS-induced ALI model. Notably, we generated miR-7 knock down mice by using miRNA-Sponge technique and found that miR-7 deficiency could ameliorate the pathologies of lung as evidenced by accelerated body weight recovery, reduced level of bronchoalveolar lavage (BAL) proinflammatory cytokines and decreased number of BAL cells in ALI mice. Moreover, the proportion and number of various immune cells in BAL, including innate immune cell F4/80^+^ macrophages, γδT cells, NK1.1^+^ T cells, and CD11c^+^DCs, as well as adaptive immune cell CD4^+^ T cells and CD8^+^ T cells, also significantly changed, respectively. Mechanistic evidence showed that KLF4, a target molecule of miR-7, was upregulated in lung tissues in ALI model, accompanied by altered transduction of NF-κB, AKT, and ERK pathway. These data provided a previously unknown role of miR-7 in pathology of ALI, which could ultimately aid the understanding of development of ALI and the development of new therapeutic strategies against clinical inflammatory lung diseases.

## Introduction

The development of acute lung injury (ALI), a clinically important complication of severe ALI in humans is a complex process, including a series of integrated steps, including the infiltration of innate and adaptive immune cells and the conversion in the balance of proinflammatory and anti-inflammatory cytokines (1–4). Recent evidence showed that microRNAs (miRNAs), small endogenous RNAs of 21–25 nucleotides capable of guiding the post-transcriptional silencing of their target mRNAs through base pairing encompassing mature mRNA 3′UTR (5, 6), played an important regulatory role in the development of ALI (7, 8). For example, Li et al. (9) reported that cardiopulmonary bypass-induced ALI could lead to dynamic changes in miRNA expression in lungs, including miR-21, miR-127, miR-145, and miR-204. Xu et al. (10) found that miR-17 expression was downregulated during development of ALI, accompanied by upregulation of its target gene FoxA1, and may play an important role in ALI. Our previously study also found that miR-155 ASO treatment could enhance the recovery of ALI through C/EBPβ, which was mediated by IL-10-secreting M2-like macrophages (Mφ) induced expansion of Tregs (11). These studies indicated that miRNAs might be a potential candidate for the therapy against ALI. Therefore, further study on the possible role of distinct miRNA molecules in the development of ALI could not only benefit the understanding of pathology of ALI but also be valuable for the development of novel clinical therapeutic strategies.

MiR-7 was a distinct miRNA family member and played an important role in the context of health, especially in organ differentiation and development (12, 13). Recent studies showed that miR-7 was involved in the development of various mammalian diseases, including lung-related diseases (14, 15). To lung cancer, accumulating evidence suggested that miR-7 was an important regulator in the development of lung cancer through controlling the growth and invasion, as well as apoptosis, of lung cancer cells and was emerged as a novel potential therapeutic target in lung cancer. Similarly, our new evidence also showed that miR-7 could regulate the proliferation and metastasis of lung cancer cells *in vitro* and *in vivo* (16). Moreover, the reduced expression of miR-7 was associated with the sites mutation of its promoter region in lung cancer tissues (17). However, the potential role of miR-7 in other lung-related diseases remains largely unknown. Interestingly, Akbas et al. (18) reported that the level of miR-7 was increased and might be a potential biomarker in chronic obstructive pulmonary disease. Moreover, Buggele et al. (19) found that influenza A virus infection could induce the expression of miR-7 in human respiratory cells, indicating miR-7 may be involved in the antivirus inflammatory reaction. Therefore, these studies raised a question regarding whether miR-7 also was involved in the development of lung-related inflammatory diseases, including ALI, which remains to be fully elucidated.

To address this aim, we generated miR-7 knock down (KD) mice by using miRNAs-Sponge technique and found that miR-7 deficiency could obviously attenuate the pathologies of lung in LPS-induced ALI mice model, which related to altered expression of related cytokines and infiltration of innate and adaptive immune cells. Of note, KLF4, a target molecule of miR-7, was upregulated in lung tissues, accompanied with reduced expression of NF-κB and phosphorylation of AKT and ERK. Thus, it is the first study showed that miR-7 played an important role in the pathology of ALI, closely correlated with upregulated expression of KLF4, which could ultimately aid the understanding of development of ALI and the development of new therapeutic strategies against clinical inflammatory lung diseases.

## Materials and Methods

### Generation of miR-7 Knock Down Mice

We annealed and ligated, gel purified and cloned oligonucleotides for miR-7 binding sites with 5-nt spacers (one spacer: 5′-CGCG-3′) for 6 bulged sites (one bulged site: 5′-AACAAAATCGAGG- -TCTTCCA-3′), into pEGFP-C2 vector (Invitrogen) digested with *Hind*III and *Kpn*I enzyme for the construction of pEGFP-C2-miR-7 sponge vector.

We next established miR-7 KD (miR-7KD) FVB/N mice using pEGFP-C2-miR-7 sponge vector with the help of Cyagen Biosciences Inc. To identify miR-7KD mice, we designed the primers spanning the EGFP sites and miR-7SP sequence, forward (P1): CGCACCATCTTCTTCAAGGA, reverse (P2): TGGAAGACCTCGATTTTGTTC. The predicted PCR product was 505 bp. All animals were housed under specific pathogen-free conditions at Zunyi Medical College. And all animal experiments were performed according to the guidelines for the Care and Use of Laboratory Animals (Ministry of Health, China, 1998). The experimental procedures were approved by the ethical guidelines of Zunyi Medical College laboratory Animal Care and Use committee (permit number 2013016).

### LPS-Induced Murine ALI

FVB/N mice (8- to 10-week-old) and miR-7KD mice (8- to 10-week-old) were challenged with i.p. injection of 10 mg/kg LPS (*Escherichia coli* 0111:B4; Sigma) dissolved in 150 μl PBS. Then the body weight of all mice was detected for 10 consecutive days. At indicated time point, the bronchoalveolar lavage (BAL) sample, lung tissue, or spleen were obtained.

### Bronchoalveolar Lavage

Immediately after euthanasia and exsanguination, 1.8 ml aliquots of PBS were slowly infused in the murine lungs through the tracheostomy and then withdrawn gently. Drainage or suction of perflubron or exudate in the airway was not performed before this lavage. This lavage was repeated four times using the same syringe. The collected lavage fluid was stored in a 2-ml tube on ice. The fluid was centrifuged at 400 × *g* and 4°C for 5 min, and the cell sediment was washed with PBS. The cell-free supernatant was centrifuged again at 16,000 × *g* and 4°C for 10 min, stored at −80°C and used for determination of cytokine content via ELISA. To the pellet, contaminating erythrocytes were lysed with 1 ml 0.15 M NH_4_Cl solution for 5 min and washed with PBS. Next, the pellet was resuspended for analysis.

### Histopathology

Lung tissue was fixed in 4% paraformaldehyde, embedded in paraffin, and cut into 4-μm-thick sections. Sections were stained with H&E, and images were taken with Olympus IX-71 microscope. Two investigators blinded to group assignments analyzed the samples and determined levels of lung injury. All lung fields at original magnification 200×, 100×, and 40× were examined for each sample.

### Flow Cytometry

Surface markers of various immune cells were evaluated by flow cytometry (FCM) with Beckman Gallios (Beckman Coulter, Inc.). FCM was performed on Beckman Gallios (Beckman Coulter, Inc.) with CellQuest Pro software using directly anti-Mouse monoclonal conjugated mAbs against the following markers: F4/80-Percp-Cy5.5 (clone: BM8; no. 45-4801-82), γδT-APC (clone: eBioGL3; no. 17-5711-81), NK1.1-APC (clone: PK136; no. 17-5941-81), CD11c-PE (clone: N418; no. 12-0114-82), CD86-APC (clone: GL1; no. 17-0862-81) and MHC-II-PE (clone: M5/114.15.2; no. 12-5321-81), CD4-Percp-Cy5.5 (clone: GK1.5; no. 12-0041-82), CD8-Percp-Cy7 (clone: 53-6.7; no. 25-0081-81), CD62L-PE (clone: MEL-14; no. 12-0621-81), CD69-APC (clone: H1.2F3; no. 17-0691-82), with corresponding isotype-matched controls (eBioscience). To detect proportion of F4/80^+^ cells, γδT^+^ cells, NK1.1^+^ cells, CD11c^+^ DC cells, CD4^+^ T cells, and CD8^+^ T cells, as well as functional molecule expression of CD86 and MHC-II on F4/80^+^ cells, and surface molecule expression of CD62L and CD69 on CD4^+^ T cells in BAL and splenocytes, cells were stained with these corresponding Abs (1:100), respectively, at 4°C for 30 min and washed twice before analysis, according to the manufacturer’s protocol.

### Real-Time PCR

The conventional primers were obtained from Shanghai Sangon Biological Engineering CO., and the TaqMan probes of miR-7 (000386) and U6 (001793) were purchased from Life Technologies, the other reagents were from TAKARA Bio Inc., Reverse transcriptase reactions and real-time PCR assays were performed according to the manufacturer’s protocols, all Reverse transcriptase reactions, including no-template controls and Reverse transcriptase minus controls, were run in triplicate in BIO-RAD CFX96 (Bio-Rad Laboratories). The expression of miR-7 was performed according to the TaqMan assays and samples were normalized by evaluating U6 expression. The other following primers were used: IL-6 forward: 5′-GGAAATCGTGGAAATGAG-3′, reverse: 5′-AGGACTCTGGCTTTGTCT-3′; IL-4 forward: 5′-AACGAGGTCACAGGAGAA-3′, reverse: 5′-CCTTGGAAGCCCTACAGA-3′; IFN-γ forward: 5′-TCTGAGACAATGAACGCTAC-3′, reverse: 5′-TTCACATCTATGCCACT-3′; KLF4 forward: 5′-GCCCAACACACACGACTTC-3′, reverse: 5′-GGCAGGA AAGGAGGGTAGTT-3′; GAPDH forward: 5′-AGCAGTCCCGTACACTGGCAAAC-3′, reverse: 5′-TCTGTGGTGATGTAAATGTCCTCT-3′. Gene expression levels were quantified using the BIO-RAD CFX96 detection system (Bio-Rad Laboratories), and the samples were normalized by evaluating GAPDH. Relative expression was calculated using the comparative threshold cycle (Ct) method.

### ELISA

The BAL level of TNF-α, IL-1β, IL-6, TGF-β, IL-10, as well as IL-4, and serum level of TNF-α, IL-10 were determined using Quantikine Immunoassay kits according to the manufacturer’s instructions, respectively (eBioscience).

### Western Blotting

Western blotting was performed on cytosolic cellular extracts. Equal amounts of protein were resolved under reducing conditions on a 10% SDS-polyacrylamide gel. Protein migration was assessed using protein standards (Bio-Rad, CA, USA). Transfer to a nitrocellulose membrane was performed 60 min at 250 mA using a wet transfer system. Equal protein loading was confirmed with Ponceau staining. The membrane was washed in 5% skim milk in PBS plus 0.05% Tween 20 (PBST) for 2 h to block non-specific protein-binding sites on the membrane. Immunoblotting was performed using an mAb to Rabbit-polyclonal anti-mouse KLF4 (Cell Signaling Technology; no. 4038), Rabbit-monoclonal anti-mouse NF-κB (Cell Signaling Technology; no. 4764), Rabbit-polyclonal anti-mouse p-NF-κB (Cell Signaling Technology; no. 3039), Rabbit-monoclonal anti-mouse ERK (Cell Signaling Technology; no. 4695), Rabbit-polyclonal anti-mouse p-ERK (Cell Signaling Technology; no. 4370), Rabbit-monoclonal anti-mouse AKT (Cell Signaling Technology; no. 4691) or Rabbit-monoclonal anti-mouse p-AKT (Cell Signaling Technology; no. 4060), Rabbit-monoclonal anti-mouse β-Actin (Cell Signaling Technology; no. 4970) at a dilution of 1/800 in a non-fat milk-Tris buffer. The membrane was then washed in PBST and subsequently probed with a secondary anti-rabbit Ab-conjugated to HRP (Cell Signaling Technology; no. 7074) at a dilution of 1:2000. The signal was detected and analyzed using the chemiluminescence Imaging System (ChemiScope5600, CLINX, Shanghai, China), each experiment was performed in triplicate.

### Immunohistochemistry

Immunohistochemical staining was performed following standard procedures. The formalin-fixed paraffin-embedded tissues were sliced into 4-μm-thick sections, deparaffinized, and rehydrated. Then, the cells were fixed with 95% ethanol for 15 min. Antigen retrieval was performed in 10 mmol/L citric acid buffer (pH 6.0) for 10 min using a 750 W microwave. Endogenous peroxidase activity was blocked with 3% hydrogen peroxide in methanol for 15 min. After incubation with rabbit anti-mouse KLF4 antibody (1:1000 dilution; CST; no. 3087) and anti-mouse NF-κB antibody (1:100 dilution; CST; no. 4764) overnight at 4°C, the sections were washed in phosphate-buffered saline and incubated with a polymer horseradish peroxidase-conjugated secondary antibody (ZSGB-Bio, Beijing, China) for 60 min. The sections were further incubated with Liquid DAB Large-Volume Substrate-Chromogen System (ZSGB-Bio, Beijing, China) and counterstained with hematoxylin. The immunostaining was evaluated using an Olympus IX-71 light microscope (Olympus, Tokyo, Japan).

### *In Situ* Hybridization

To evaluate the cellular distribution of miR-7 in the lungs, *in situ* hybridization was performed based as previously described (20) with some modifications. Briefly, before hybridization incubation, all solutions were prepared with diethyl pyrocarbonate-treated water. After deparaffinization and rehydration, tissue sections were treated by proteinase K digestion. After blocking with normal goat serum (1:100), sections were next incubated or microwave heating and were then incubated with hybridization cocktail containing miR-7 probe (1:1000 dilution; EXIQON; no. 38485-01) at 42°C for 16 h. Then alkaline phosphatase-labeled anti-digoxigenin antibody (1:500) (Roche Diagnostics) for 1 h, and the reaction products were colorized with nitro blue tetrazolium/5-bromo-4-choloro-3-indolyl phosphate (NBT/BCIP) (ZSGB-Bio, Beijing, China), then the tissues were counterstained with Mayer’s hematoxylin and systematically viewed under a light microscope (Olympus IX-71).

### Statistical Analyses

The data were analyzed with GraphPad Prism 5.0 and were presented as the mean ± SD. Student’s *t*-test was used when two conditions were compared, and analysis of variance with Bonferroni or Newman–Keuls correction was used for multiple comparisons. Probability values of <0.05 were considered significant; two-sided tests were performed.

## Results

### Generation of miR-7 Knock Down Mice

To investigate the potential role of miR-7 in the development of ALI, we detected the relative expression level of miR-7 in murine 12 organs, including heart, lung, brain, kidney, and so on. Data showed that miR-7 expression had a higher level in lung tissue (Figure S1A in Supplementary Material). Expectedly, we further found that the expression of miR-7 significantly increased in pathogenic lung tissues in LPS-induced ALI mice (Figure S1B in Supplementary Material, *p* < 0.05). Together, these data indicated that miR-7 might be involved in the development of ALI.

**Figure 1:**
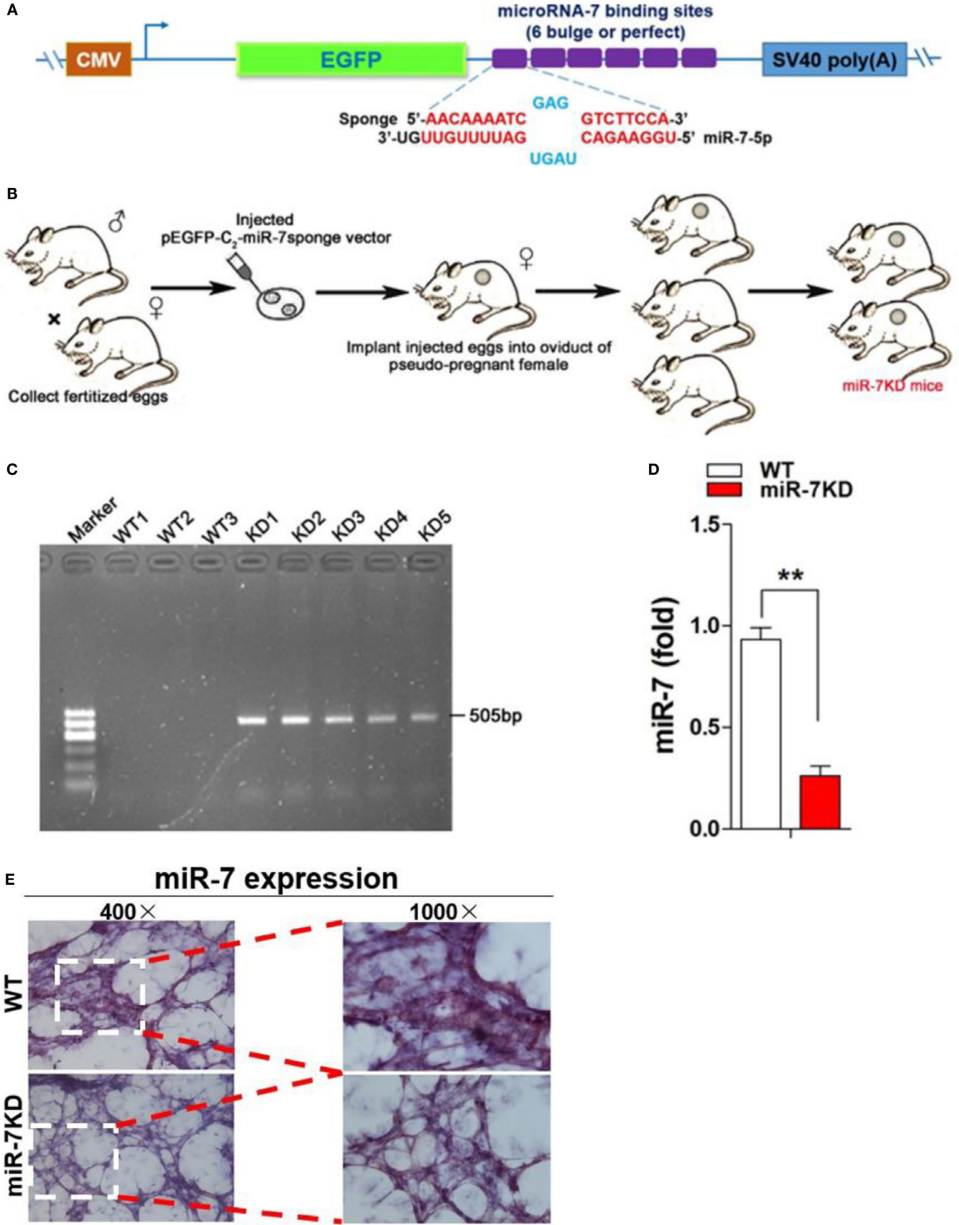
**The generation of miR-7 deficiency mice**. The sketch map of construction of eukaryotic expression vector encoding miR-7-Sponge (termed as p-miR-7-Sp) **(A)** and generation of miR-7 KD mice **(B)**. **(C)** The identification of DNA. DNA was derived from wild-type (WT) or miR-7KD mice. And the expected product (505 bp size) was amplificated by PCR, respectively. **(D)** The relative expression of miR-7 in lung derived from WT mice and miR-7KD mice (*n* = 6) were detected by Real-time PCR assay. **(E)** The expression of miR-7 in lung tissue derived from WT mice and miR-7KD was analyzed by *In situ* hybridization. Representative data of three independent experiments were shown. **p < 0.01.

Accumulating evidence showed that miRNA Sponge strategy was valuable approach for the downregulation of specific miRNA expression *in vivo* (21, 22). Moreover, it is difficult to generate miR-7 deficiency mice using traditional strategies, including TALENs and CRISPR, because of mature miR-7 molecule is transcribed from three different genomic loci on chromosomes 9, 15 and 19, respectively (23). Then, we used miRNA Sponge (miR-SP) strategy to generate mice with miR-7 deficiency. The design and proof of principle of miR-SP technology is to construct a eukaryotic expression vector encoding an expression cassette for miR-7-SP sequence, the miR-7-SP elements included inserting tandemly arrayed miR-7 binding sites into the 3′UTR of a reporter gene encoding destabilized EGFP driven by the CMV promoter. Binding sites for miR-7 seed family were perfectly complementary in the seed region with a bulge at positions 9–12 to prevent RNA interference-type cleavage and degradation of the sponge RNA, the bulge contained six repetitive sequences complementary to miR-7 with mismatches for enhanced stability (Figure 1A). Then, transgenic mice were generated carrying one or two copies of the miR-SP cassette through putting the miR-7SP carrier to inject murine fertilized eggs, after which were transplanted into the oviduct of pseudo-pregnant mice female, and 3 weeks after birth (Figure 1B). To verify a special KD of miR-7 in FVB/N mice, we designed primers (P1 and P2) spanning the EGFP sites and miR-7SP sequence, and the predicted PCR product size was 505 bp. Then, we detected both wild-type (WT) mice and miR-7KD mice, while only miR-7KD mice could amplify 505 bp specific products (Figure 1C). Importantly, we found that the relative expression of miR-7 was obviously reduced in lung in miR-7KD mice (Figure 1D, *p* < 0.05). To confirm these data, we further detected the expression of miR-7 in lung tissues of miR-7KD mice using *in situ* hybridization and obtained similar results (Figure 1E). Consistently, the relative expression of miR-7 in other various organs and tissues in miR-7KD mice also decreased significantly (Figure S2 in Supplementary Material, *p* < 0.05). These results demonstrated that miR-7KD mice were successfully generated using miR-SP Technology.

### Deficiency of miR-7 Ameliorated the Pathologies of ALI

**Figure 2:**
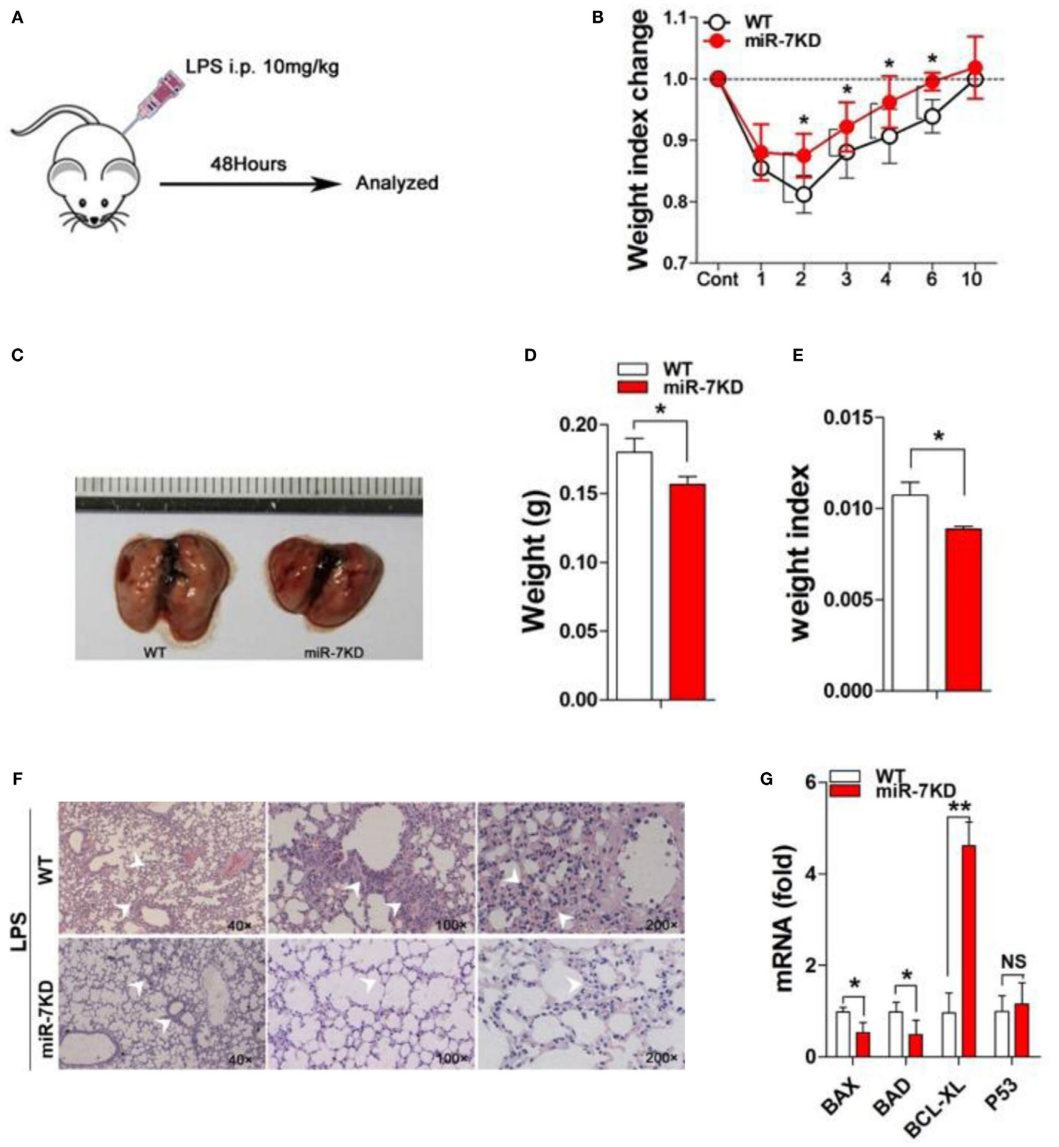

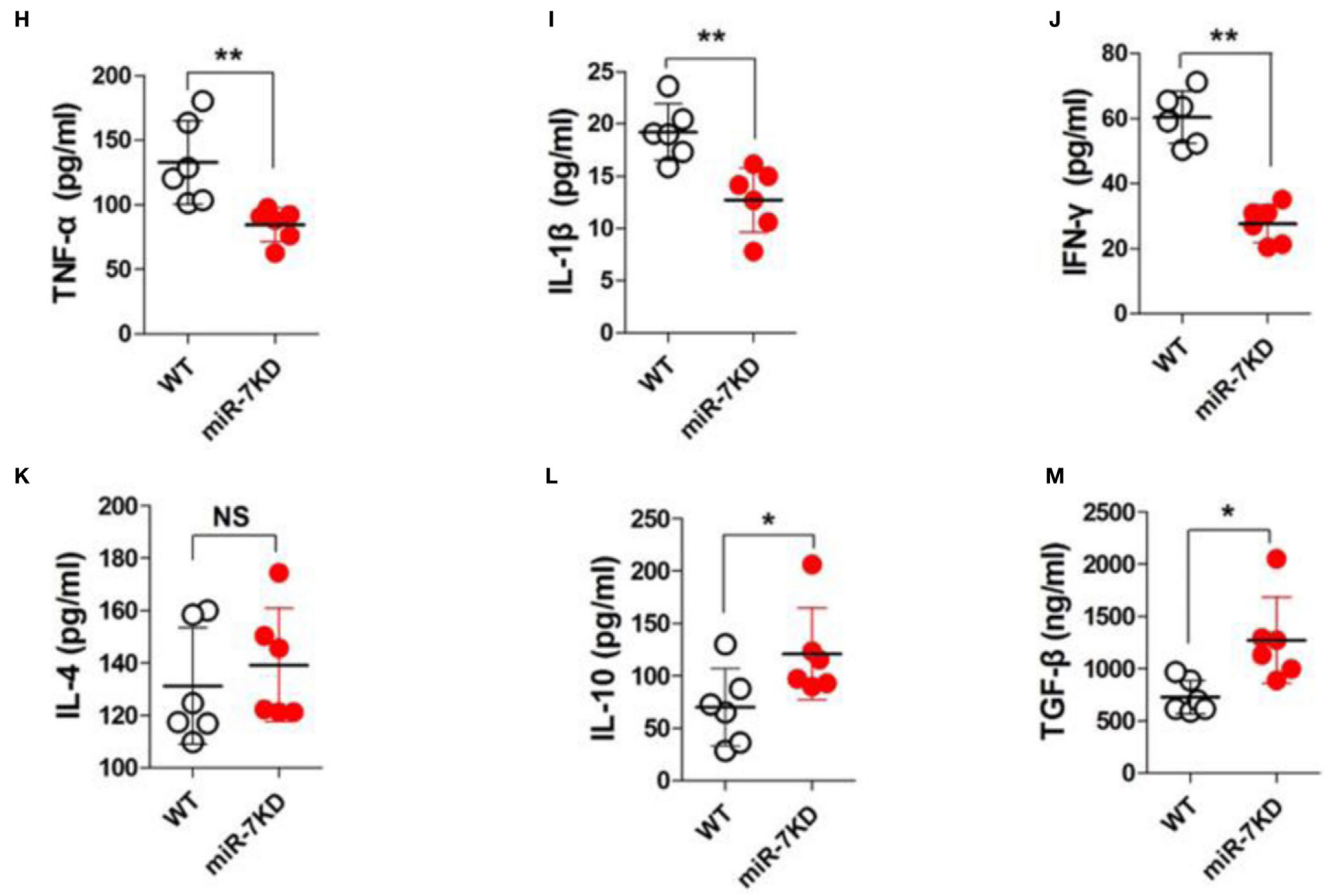
**miR-7 deficiency ameliorated the pathology of ALI**. FVB/N6 mice (WT, *n* = 6) and miR-7KD mice (*n* = 6) were administered with i.p. 10 mg/kg LPS, respectively. **(A)** Schematic representation of the animal study. **(B)** The change on the body weight of WT and miR-7KD mice at indicated time point was observed. **(C)** FVB/N6 mice (WT, *n* = 6) and miR-7KD mice (*n* = 6) were administered with i.p. 10 mg/kg LPS, respectively. After 48 h, the morphology, **(D)** the body weight, and **(E)** the weight index (tissue weight/body weight), as well as **(F)** the pathology, of lung tissues were analyzed, respectively. **(G)** The relative expression of BAD, BAX, BCL-XL, and p53 in lung tissue also were analyzed by Real-time PCR assay. **(H–M)** FVB/N6 mice (WT, *n* = 6) and miR-7KD mice (*n* = 6) were administered with i.p. 10 mg/kg LPS, respectively. After 48 h, BAL was collected and the level of IFN-γ, IL-1β, TNF-α, IL-4, and IL-10, as well as TGF-β, was determined with ELISA, respectively. Representative data of three independent experiments were shown. *p < 0.05, **p < 0.01.

Next, we observed the possible effects of miR-7 deficiency on the development of ALI using a murine model of LPS-induced ALI. Results showed that the loss of body weight was attenuated in LPS-treated miR-7KD group, compared with that in LPS-treated WT group (Figures 2A,B, *p* < 0.05). Importantly, the morphology of lung tissue of mice in LPS-treated miR-7KD group was smaller than that in LPS-treated WT group (Figure 2C). Furthermore, the weight and the weight index of lung tissue were also significantly reduced (Figures 2D,E, *p* < 0.05). Meanwhile, hematoxylin-eosin staining showed that the infiltration of inflammatory cells in lung tissue of miR-7KD ALI mice markedly decreased (Figure 2F). The cell apoptosis of lung tissue was involved in the pathology of lung injury (24, 25). As shown in Figure 2G, real-time PCR analysis further showed that the expression of anti-apoptotic proteins BCL-XL in lung tissue increased significantly in LPS-treated miR-7KD mice (*p* < 0.05), even though the expression of p53 did not change significantly (*p* > 0.05). Conversely, the expression of pro-apoptotic proteins BAX and BAD were decreased obviously (*p* < 0.05), indicating the cell apoptosis of lung tissue decreased in miR-7KD ALI mice.

To confirm this phenomenon, we further detected the level of related cytokines in ALI mice. As shown in Figures 2H–J, the concentration of proinflammatory factor TNF-α, IL-1β, and IFN-γ in BAL in LPS-treated miR-7KD group decreased significantly compared with those in the control group on 3 days (*p* < 0.05). By contrast, the concentration of anti-inflammatory factor IL-10 and TGF-β increased noticeably (*p* < 0.05), even though the concentration of IL-4 in BAL did not change significantly (Figures 2K–M, *p* > 0.05). To verify these data, we detected the relative expression of these cytokines in lung tissues, and similar results were obtained (Figure S3 in Supplementary Material). Finally, we also detected the serum level of cytokines TNF-α and IL-10, respectively. Data showed that the serum level of TNF-α decreased in LPS-treated miR-7KD mice (Figure S4A in Supplementary Material, *p* < 0.05). Conversely, the serum level of IL-10 increased significantly (Figure S4B in Supplementary Material, *p* < 0.05). Combining these data demonstrated that miR-7 deficiency could obviously ameliorate the pathologies of ALI.

### miR-7 Deficiency Altered the Composition of Immune Cells in BAL of ALI Mice

**Figure 3:**
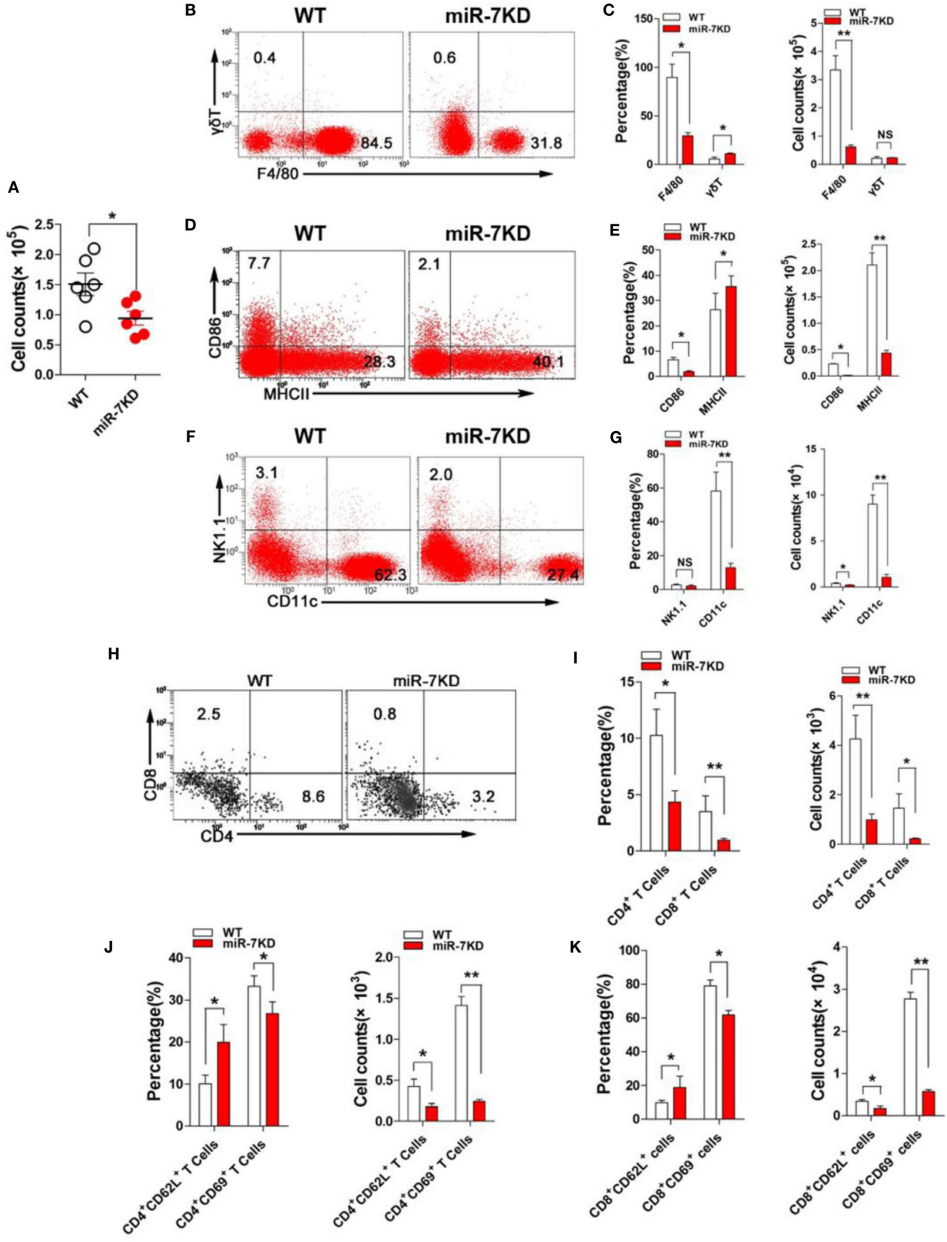
**miR-7 deficiency altered the immune cell composition in BAL of mice from ALI**. FVB/N6 mice (WT, *n* = 6) and miR-7KD mice (*n* = 6) were administered with i.p. 10 mg/kg LPS, respectively. After 48 h, **(A)** the total numbers of BAL cells were calculated. **(B)** The proportion of F4/80^+^ Mφ and γδ^+^ T cells were analyzed by FCM. The percentage and the absolute number of cells were calculated, respectively **(C)**. **(D)** The expression of CD86 and MHC-II on F4/80^+^ Mφ were analyzed by FCM. The percentage and the absolute number of cells were calculated, respectively **(E)**. **(F)** The proportion of CD11c^+^ dendritic cells and NK1.1^+^ T cells were analyzed by FCM. The percentage and the absolute number of cells were calculated, respectively **(G)**. **(H)** The proportion of CD4^+^ T cells and CD8^+^ T cells were analyzed by FCM. The percentage and the absolute number of cells were calculated, respectively **(I)**. **(J)** The expression of CD62L and CD69 on CD4^+^ T cells were analyzed by FCM. And, the percentage and the absolute number of cells were also calculated, respectively. **(K)** The expression of CD62L and CD69 on CD8^+^ T cells were analyzed by FCM. And, the percentage and the absolute number of cells were also calculated, respectively. Representative data of three independent experiments were shown. *p < 0.05, **p < 0.01.

The change of immune cell composition, which was closely related to inflammatory responses, plays a pivotal role in the pathology of ALI (26, 27). Hence, we examined the total number cells in BAL from LPS-treated WT and miR-7KD mice. As shown in Figure 3A, the total number of inflammatory cells in BAL from miR-7KD ALI mice was significantly decreased (*p* < 0.05). We further analyzed the possible changes on proportion of the innate immune cells, including F4/80^+^ Mφ, γδT cells, NK1.1^+^ T cells, CD11c^+^ Dendritic cells (DCs) in BAL from miR-7KD ALI mice and found that the proportion and the absolute number of F4/80^+^ Mφ and CD11c^+^ DCs obviously reduced in BAL (Figures 3B,C,F,G, *p* < 0.05). Unexpectedly, the absolute number of γδT cells in BAL did not change significantly (Figures 3B,C, *p* > 0.05), even though the proportion of γδT cells increased (Figures 3B,C, *p* < 0.05). Meanwhile, we also investigated the possible change on adaptive immune cells CD4^+^ T cells and CD8^+^ T cells, which also played important roles in the development of ALI, in BAL of LPS-treated miR-7KD mice. The results showed that the proportion and the absolute number of CD4^+^ T cells and CD8^+^ T cells are also obviously reduced in BAL (Figures 3H,I, *p* < 0.05). To confirm this phenomenon, we also observed the total number, the proportion, and the absolute number of the innate immune cells, including F4/80^+^ Mφ, γδT cells, NK1.1^+^ T cells, CD11c^+^ DCs, and adaptive immune cells, including CD4^+^ T cells and CD8^+^ T cells in the spleen. Expectedly, similar results also were obtained (Figures S5A–C,F–I in Supplementary Material).

As known, the expression of the functional membrane molecules on immune cells was closely related to the pathological status of inflammatory diseases (28); hence, the expression of CD86 and MHC-II molecule in F4/80^+^ Mφ were analyzed. FCM analysis showed that the proportion and absolute number of CD86 positive F4/80^+^ Mφ obviously reduced. And the absolute number of MHC-II positive F4/80^+^ Mφ also decreased significantly in BAL, even though the proportion of cells increased (Figures 3D,E). Next, we further analyzed the expression of activation molecules CD62L and CD69, respectively, in both CD4^+^ T cells and CD8^+^ T cells. Data showed that the expression of CD62L on CD4^+^ T cells and CD8^+^ T cells were significantly upregulated (Figures 3J,K, *p* < 0.05). Conversely, the expression of CD69 on CD4^+^ T cells and CD8^+^ T cells were significantly downregulated (Figures 3J,K, *p* < 0.05), indicating reduced activation of CD4^+^ T cells and CD8^+^ T cells. Consistent with above data, the absolute number of these cells were also reduced vigorously (Figures 3J,K, *p* < 0.05). Similarly, we also obtained the consistent results in the spleen from LPS-treated miR-7KD mice (Figures S5D,E,J,K in Supplementary Material). In summary, our results demonstrate that miR-7 deficiency could significantly affect the composition and function of various immune cells in BAL, which was closely related to ameliorated pathology of lung in ALI mice.

### The Expression of KLF4 Was Upregulated in Lung Tissues in LPS-Treated miR-7KD Mice

**Figure 4:**
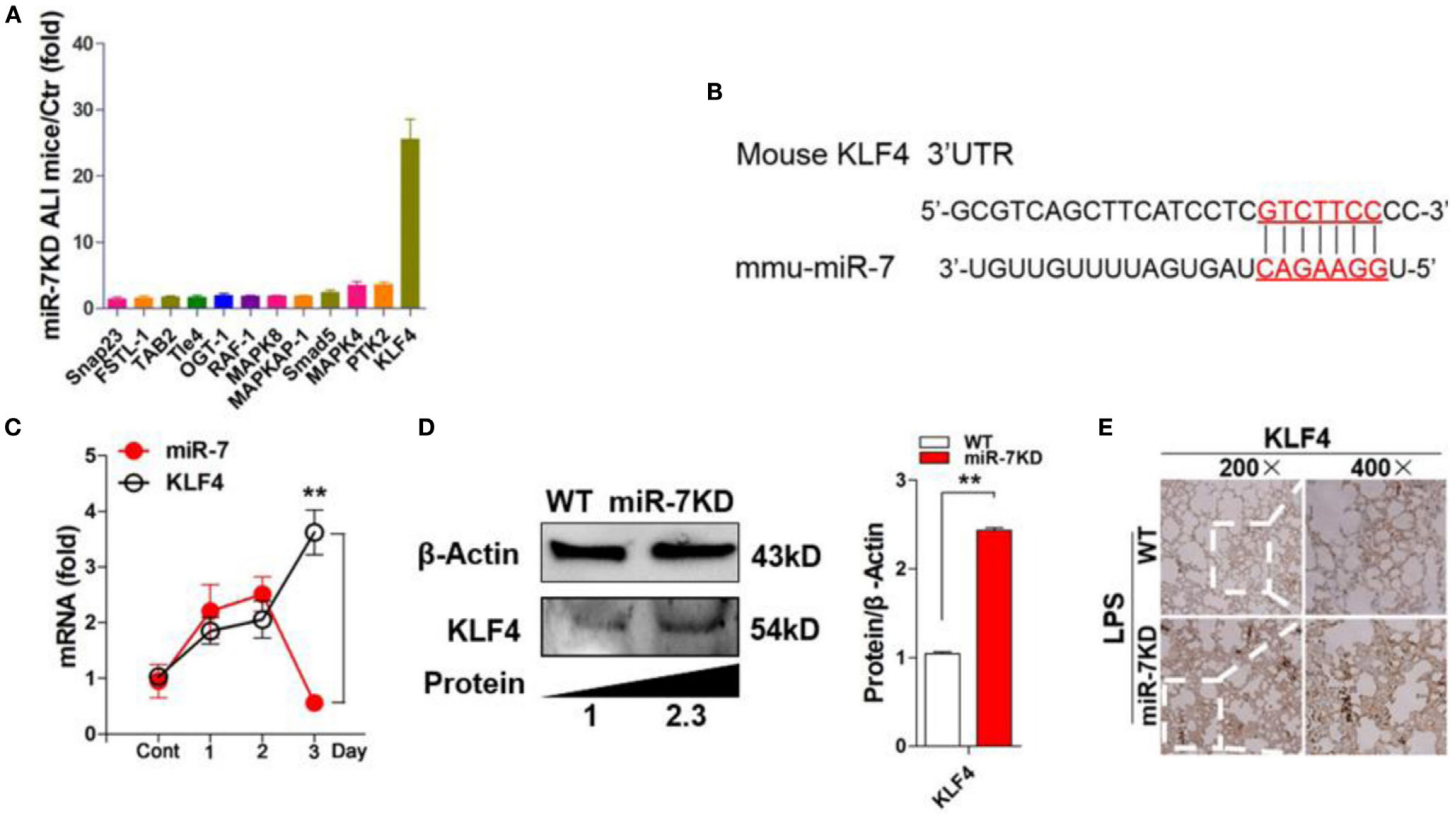
**KLF4 was upregulated in lung tissues in LPS-treated miR-7KD mice**. **(A)** FVB/N6 mice (WT, *n* = 6) and miR-7KD mice (*n* = 6) were administered with i.p. 10 mg/kg LPS, respectively. After 48 h, the relative expression of the potential target genes of miR-7 in the lung tissue were analyzed by Real-time PCR assay. **(B)** Putative miR-7-binding sites in the 3′UTR of KLF4. **(C)** FVB/N6 mice (WT, *n* = 6) were administered with i.p. 10 mg/kg LPS. The relative expression of miR-7 and KLF4 were detected by Real-time PCR at indicated time points. **(D)** FVB/N6 mice (WT, *n* = 6) and miR-7KD mice (*n* = 6) were administered with i.p. 10 mg/kg LPS, respectively. After 48 h, KLF4 expression in lung tissue was analyzed by Western Blot and **(E)** immunohistochemical staining, respectively (original magnification 200×, 400×). Representative data of three independent experiments were shown. *p < 0.05, **p < 0.01.

To elucidate the potential molecular mechanism through which miR-7 deficiency affected the pathologies of lung injury, we searched for putative targets of miR-7 using computer-aided miRNA target prediction program, including TargetScan4, miRanda, and PicTar, and found 12 putative miR-7 target genes, including Snap23, TAB2, KLF4, Smad5, MAPK4, FSTL-1, Tle-4, OGT-1, RAF-1, MAPK8, MAPKAP-1, and PTK2, which also were closely associated with inflammation reaction according to previous literatures (29–34). Then, we detected the expression of these 12 predicted target genes in the lung tissues derived from LPS-treated WT mice and miR-7KD mice, respectively. Unexpectedly, Real-time PCR assay showed that only one target, KLF4, in all predicted target genes of miR-7 was significantly upregulated by more than 25-fold in the lung tissue of LPS-treated miR-7KD mice compared with LPS-treated WT mice (Figures 4A,B).

Recent studies showed that KLF4, a critical regulator in LPS-induced inflammatory response (35), was one of targets of miR-7 (36). Moreover, KLF4 also could promote the expression of anti-inflammatory cytokines, such as IL-10 (37). Then, to investigate the potential connection between miR-7 and KLF4 in lung injury in ALI, we further detected the relative expression of miR-7 and KLF4 in LPS-treated WT mice at different time points. As shown in Figure 4C, the relative expression of miR-7 increased significantly on day 1, and reached the peak on day 2, then decreased on day 3. Consistently, the relative expression of KLF4 decreased obviously in day 2 and increased significantly on day 3. Next, to verify the role of KLF4 in the effect of miR-7 deficiency on the pathology of ALI, we further detected the protein level of KLF4 in lung tissues from LPS-treated miR-7KD mice and WT mice. As shown in Figure 4D, the level of KLF4 protein in lung tissue was increased significantly in LPS-treated miR-7KD mice compared with that in LPS-treated WT mice (*p* < 0.05). To confirm these data, we further observed the location of KLF4 protein in the injured lung tissue. As anticipated, we found strong KLF4 protein staining on lung tissue derived from miR-7KD ALI mice compared with WT ALI mice (Figure 4E). Collectively, our data indicated that miR-7 deficiency could affect the pathology of ALI, which was closely due to the upregulation expression of its target KLF4.

### miR-7 Deficiency Altered the Transduction of Related Signaling Pathway

**Figure 5:**
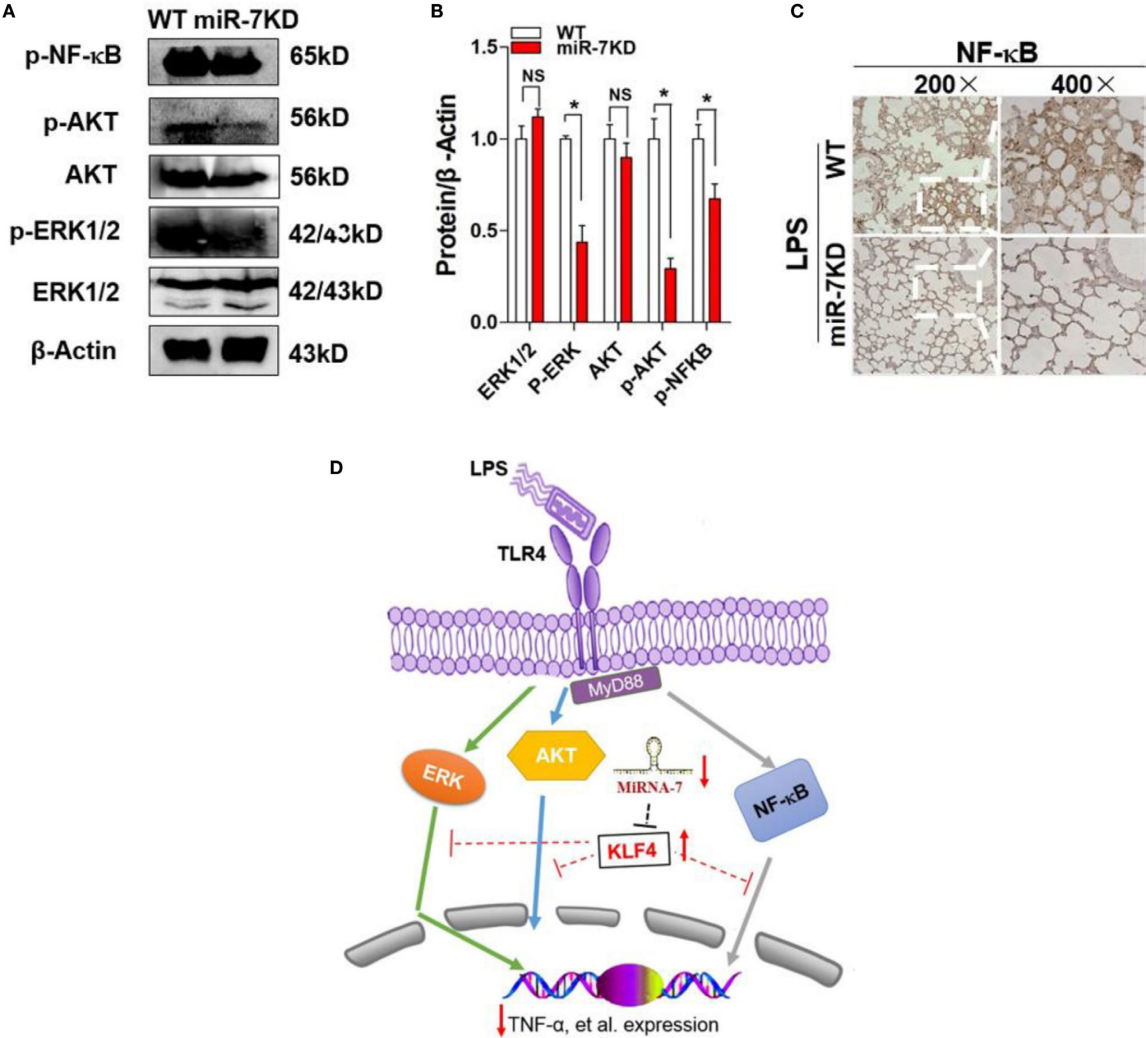
**The signaling pathway change of miR-7 deficiency in ALI**. FVB/N6 mice (WT, *n* = 6) and miR-7KD mice (*n* = 6) were administered with i.p. 10 mg/kg LPS, respectively. After 48 h, the expression level of ERK, p-ERK, AKT, p-AKT, and p-NF-kB in lung tissue were determined with Western Blot **(A)** and calculated **(B)**. **(C)** The expression of NF-κB was also detected by the immunohistochemical staining. Representative data of three independent experiments were shown. *p < 0.05, **p < 0.01. **(D)** Schematic representation of the underlying mechanism of miR-7 deficiency on ALI. miR-7 deficiency leads to upregulation of KLF4, which could successively alter the transduction of NF-κB, ERK, and AKT signaling pathway and contribute to the ameliorated pathologies of ALI.

The multiple signaling pathways, including AKT, ERK, and so on, were also involved in the development process of ALI (38). Moreover, KLF4 also was reported to be closely related to the transduction of these signaling pathways (39). Thus, to elucidate whether miR-7 deficiency resulted in the attenuated pathologies of ALI might be related with these signaling pathway, the expression of phosphorylation of AKT and ERK were analyzed in lung tissue derived from LPS-treated WT mice or miR-7KD mice, respectively. Data showed that there were not any change on the expression level of AKT and ERK in between miR-7KD ALI mice and WT ALI mice (Figures 5A,B, *p* > 0.05). However, the phosphorylation of AKT and ERK in miR-7KD ALI mice decreased significantly, compared with those in WT ALI mice (Figures 5A,B, *p* < 0.05).

Furthermore, we also detected the expression of NF-κB, which was critical transcription factor for inflammatory reaction, in lung tissue from miR-7KD ALI mice. Data showed that the expression of phosphorylation of NF-κB also decreased obviously (Figures 5A,B, *p* < 0.05). To confirm this phenomenon, we further analyzed the expression of phosphorylation of NF-κB in lung tissue using immunohistochemical staining and similar result was obtained (Figure 5C). These results suggested that the evident effect of miR-7 deficiency on the pathology of ALI was closely correlated to the altered transduction of NF-κB and AKT, as well as ERK, signaling pathway (Figure 5D).

## Discussion

Up to now, this is the first study to explore the potential role of miR-7 in the pathologies of ALI. We, first, observed that the expression of miR-7 increased significantly in lung tissue in murine LPS-induced ALI model. Furthermore, we generated miR-7 deficiency mice by using miR-SP technology. Importantly, we found that miR-7 deficiency could significantly attenuate the pathologies of ALI, evidenced by accelerated body weight recovery, reduced infiltration of BAL cells and altered level of related cytokines, which was closely correlated to upregulation of KLF4, a target of miR-7, and altered transduction of related signaling pathway, including NF-κB, AKT, and ERK pathway.

Accumulating evidence suggested that miR-SP, which are transcripts that carry several in tandem copies of a sequence complementary to the miRNA of interest in their 3′UTRs, was a promising approach for loss-of-function strategies of distinct miRNA molecule *in vivo* (40, 41). Giusti et al developed a transgenic miRNA sponge mouse for investigation on the biological function of miR-9 in dendritic growth and synaptic transmission *in vivo* (42). Most recently, Zheng et al. constructed “Sponge” transgenic mice against miR-27a expression and found that Siglec1 and TRIM27 expression, target molecules of miR-27a, were elevated *in vivo* in antiviral innate response (43). In the current study, we generated miR-7 deficiency mice using miR-SP technology. Our data showed that the relative expression of miR-7 in various organs, including lung, spleen, brain, and so on, dramatically decreased, indicating miR-7-SP could effectively inhibit the expression of miR-7 *in vivo*. Importantly, we further found that miR-7 deficiency could obviously ameliorate the development of LPS-induced ALI, evidenced by prevention of body weight loss and reduced weight and pathological change of lung tissue. Moreover, our data further showed that, miR-7 not only was enriched in a variety of normal tissues, including brain and lung tissue, which was consistent with previous literature (44–46), but also was upregulated in lung tissue of LPS-induced ALI model. Similarly, Akbas et al. (18) reported that the level of miR-7 was increased and might be a potential biomarker in chronic obstructive pulmonary disease. These data might reflect the important role of miR-7 in the development of lung-related diseases, including ALI. Therefore, successive research work on the expression of miR-7 in clinical ALI cases, as well as the potential effect of miR-7 on other animal ALI models that were with distinct traits related to diverse pathological characters of clinical ALI, is much valuable for the validation on the role of miR-7 in pathology of ALI.

The infiltration of innate immune cells and adaptive immune cells, including Mφ, γδ^+^ T cells, and CD4^+^ T cells, were closely involved in the development process of ALI (47). For example, Holub and Lawrence (48) reported that the proportion of Mφ in BAL increased significantly and was contributed to the pathology of ALI. In our study, we found that the proportion and the cell absolute number of not only innate immune cells, such as F4/80^+^ Mφ, but also adaptive immune cells, including CD4^+^ T cells and CD8^+^ T cells, in BAL also decreased significantly. Moreover, similar results also were obtained in splenocytes. Notably, we further reported that altered expression of activated membrane molecular, including CD86 and MHC-II, on F4/80^+^ Mφ cells in LPS-treated miR-7 KD mice. Meanwhile, CD4^+^ T cells and CD8^+^ T cells also expressed lower level of CD69 and higher level of CD62L, displaying a decreased activation phenotype. Similarly, Ying et al. (49) reported that downregulation of miR-127 could impair the infiltration and function of Mφ, which was contributed to the decreased pathology of ALI. Moreover, Brusselle et al. (50) also found that the dysregulation of miRNAs could contribute to the pathogenesis of lung-associated inflammatory diseases, which were related to the impaired activation of CD4^+^ T cells in ALI. Combining these data suggested that the change on infiltration and functional molecule of these immune cells are contributed to impaired pathologies of ALI in miR-7 KD mice. We proposed that two factors might be responsible for the change on infiltration of immune cells. First, the altered cytokine balance was important for the infiltration of immune cells because various cytokines, such as IL-10, TNF-α, and so on, could affect the migration of immune cells. Second, the potential role of miR-7 in biological function of immune cells, even was still unknown, also might be contributed to the altered migration of immune cells. It is interesting to note that recent research works showed the important role of distinct miRNA molecules in biological function of immune cells (11, 51). Therefore, our current data might provide valuable clues for the related research work on the potential function of miR-7 in immune cells, which also should be helpful for understanding the role of miR-7 in development of ALI.

It is well known that some signaling pathways, such as NF-κB, AKT, and ERK pathway, were critical for the development of inflammatory response (52–54). And KLF4, as one putative target of miR-7, played a critical role in the transduction of these signaling pathways, which were involved in the development of various diseases (55–59). To ALI, genetic analysis suggested that KLF4 was one of the leading candidate genes associated with increased susceptibility to ALI (60). And KD of KLF4 obviously augmented LPS-induced lung injury (61). In the present study, we found that the expression level of KLF4 increased in lung tissue in LPS-treated miR-7KD mice. Importantly, we further found that the transduction of NF-κB and AKT pathway, as well as ERK pathway, decreased obviously. Moreover, the level of proinflammatory cytokines, such as TNF-α, IL-1β, and IL-6, also dramatically decreased, and the level of anti-inflammatory cytokines, such as IL-10 and TGF-β1, conversely increased. Consistently, one most recent research work reported that KLF4 could potentially regulate the transduction of NF-κB pathway, which closely related to decreased levels of the pro-inflammatory cytokines TNF-α (62). Moreover, KLF4 also could bind to IL-10 and TGF-β1 promoter, as a transcriptional regulator, and upregulate the transcriptional level of IL-10 and TGF-β1, respectively (37, 63). Therefore, given the fact that regulation effect of miR-7 on KLF4 expression, these data further highlighted the critical role of miR-7/KLF4 axis in development of ALI, indicating the potential value of this axis in the development of therapeutic strategy against inflammatory lung diseases.

Taken together, as shown in Figure 5D, we presumed that miR-7 deficiency altered that expression of KLF4, which affected the transduction of related signaling pathway, such as NF-κB, AKT, and ERK, and successively influence on the infiltration of various immune cells and the level of inflammatory cytokines, which ultimately ameliorated the pathologies of LPS-induced ALI.

## Author Contributions

J Zhao and CC performed the experiments, analyzed the data, and wrote the paper; MG and YT performed the experiments and analyzed the data; PC, NQ, and J Zheng performed the experiments; YZ and J Zhang wrote the paper; LX conceived and designed the experiments, analyzed the data, and wrote the paper; and all authors reviewed the paper.

## Conflict of Interest Statement

The authors declare that the research was conducted in the absence of any commercial or financial relationships that could be construed as a potential conflict of interest. The reviewer SM-J and handling editor declared their shared affiliation, and the handling editor states that the process nevertheless met the standards of a fair and objective review.
