# Supplementary material for: MicroRNA-7 Deficiency Ameliorates the Pathologies of Acute Lung Injury through Elevating KLF4
Source: Front Immunol. 2016 Oct 7;7:389. doi: 10.3389/fimmu.2016.00389 (PMC5054040; doi:10.3389/fimmu.2016.00389)
Supplement: Supplementary file 1 [file Presentation_1.PDF]

Supplementary Material

MicroRNA-7 deficiency ameliorates the pathologies of Acute Lung Injury through Elevating KLF4

Juanjuan Zhao[1,+], Chao Chen[1,+], Mengmeng Guo[1], Yijin Tao[1], PanPan Cui[1], Ya Zhou[2], Nalin Qin[1], Jing Zheng[1], Jidong Zhang[1], Lin Xu[1,*]

[1] Department of Immunology, Zunyi Medical College, Guizhou, 563000 China

[2] Department of Medical physics, Zunyi Medical College, Guizhou, 563000 China

[*] **Correspondence to:**

Dr. Lin Xu, Department of immunology, Zunyi Medical College, GuiZhou Province, 563003 P.R.China, Email: xulinzhouya@163.com

[+] Juanjuan Zhao and Chao Chen contributed equally to this work.

Data of supplements: 5

Supplementary Figure 1

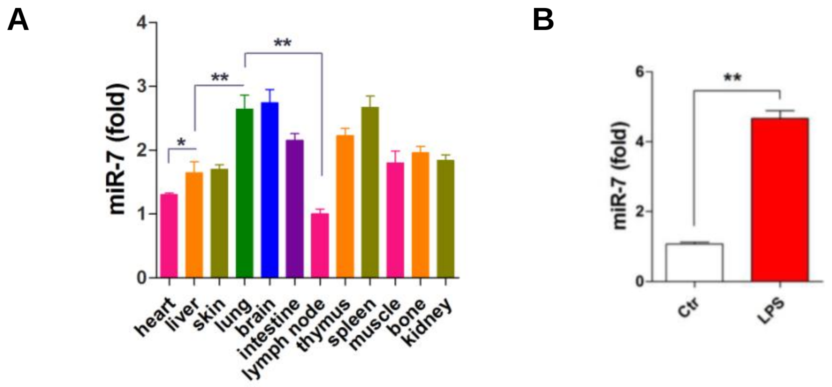

Supplementary Figure 1 The relative expression of miR-7.

(A) The indicated 12 organs and tissues (heart, liver, skin, lung, brain, intestine, lymph node, thymus, spleen, muscle, bone and kidney) were collected from normal FVB/N6 mice (n=6). The relative expression of miR-7 was detected by Realtime PCR assay and fold change was normalized to lymph node respectively. (B) FVB/N6 mice (n=6) were administered with i.p. 10 mg/kg LPS, after 48 hrs later, the lung tissue were harvested and the relative expression of miR-7 was analyzed by Realtime PCR. **$p<0.01$. *$p<0.05$.

.

Supplementary Figure 2

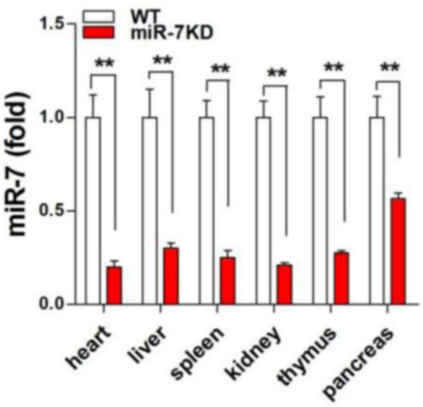

Supplementary Figure 2 The relative expression of miR-7 in the multiple organs of miR-7KD mice model.

The relative expression of miR-7 in 6 organs (heart, liver, spleen, kidney, thymus, and pancreas) derived from miR-7KD mice (n=6) were detected by Realtime PCR assay respectively. **p<0.01.

Supplementary Figure 3

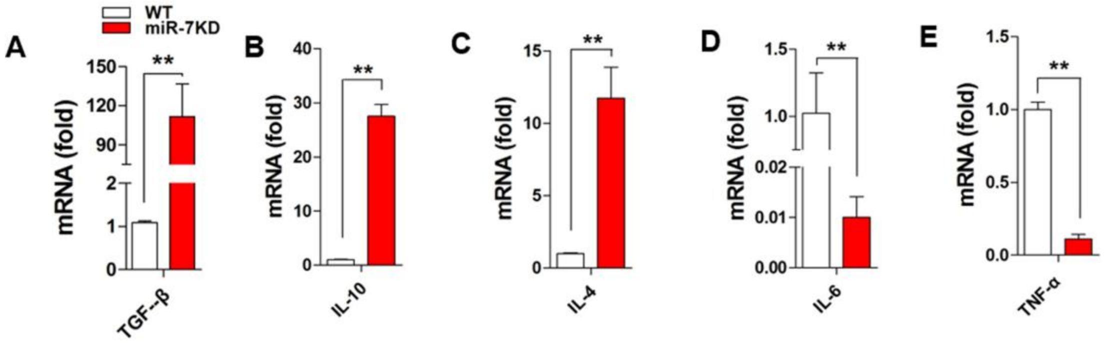

Supplementary Figure 3 The relative expression of cytokines in lung tissues in LPS-treated miR-7KD mice.

miR-7KD mice (n=6) and WT mice (n=6) were i.p. injected with 10 mg/kg LPS. After 48 hours later, the lung tissues were harvested and the relative expression of anti-inflammatory cytokines (A) TGF-β, (B) IL-10 and (C) IL-4, as well as proinflammatory cytokines (D) IL-6 and (E) TNF-α were detected by Realtime PCR assay respectively. Data are representative of three independent experiments. ** $p<0.01$.

Supplementary Figure 4

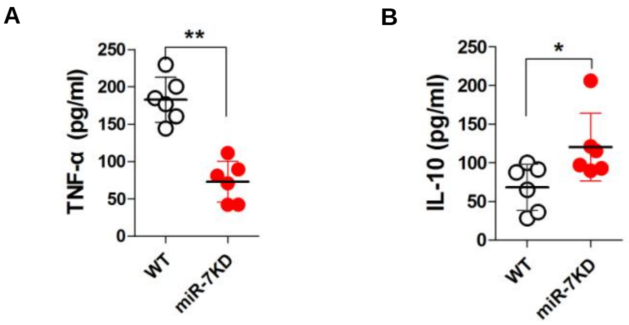

Supplementary Figure 4 The serum level of IL-10 and TNF-α in LPS-induced ALI.

miR-7KD mice (n=6) and WT mice (n=6) were i.p. injected with 10 mg/kg LPS. After 48 hrs later, the serum level of (A) TNF-α and (B) IL-10 were determined with ELISA. One representative data of three independent experiments was shown. *$p<0.05$, **$p<0.01$.

Supplementary Figure 5

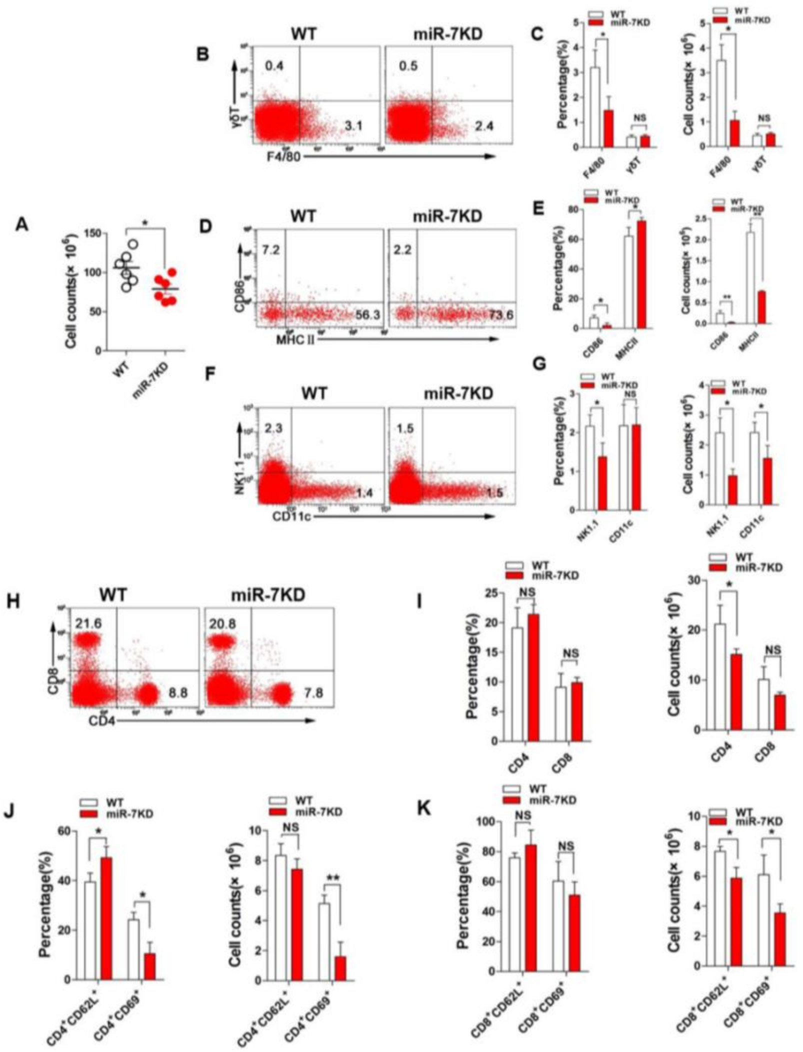

Supplementary Figure 5 miR-7 deficiency altered the immune cell composition in Spleen of ALI mice.

FVB/N6 mice (WT, n=6) and miR-7KD mice (n=6) were administered with i.p. 10 mg/kg LPS respectively. 48 hrs later, (A) The total numbers in splenocytes were calculated. (B) The proportion of F4/80$^+$ macrophages and $\gamma\delta^+$ T cells were analyzed by FCM. The percentage and the absolute number of cells were calculated respectively (C). (D) The expression of CD86 and MHC-II on F4/80$^+$ macrophages were analyzed by FCM. The percentage and the absolute number of cells were calculated respectively (E). (F) The proportion of CD11c$^+$ dendritic cells and NK1.1$^+$ T cells were analyzed by FCM. The percentage and the absolute number of cells were calculated respectively (G).

(H) The proportion of $CD4^+$ T cells and $CD8^+$ T cells were analyzed by FCM. The percentage and the absolute number of cells were calculated respectively (I). (J) The expression of CD62L and CD69 on $CD4^+$ T cells were analyzed by FCM. And, the percentage and the absolute number of cells were also calculated respectively. (K) The expression of CD62L and CD69 on $CD8^+$ T cells were analyzed by FCM. And, the percentage and the absolute number of cells were also calculated respectively. One representative data of three independent experiments was shown. $*p<0.05$, $**p<0.01$.
